# Concentrated pineapple juice for visualisation of the oesophagus during magnetic resonance angiography before atrial fibrillation radiofrequency catheter ablation

**DOI:** 10.1186/s41747-018-0067-0

**Published:** 2018-11-21

**Authors:** Riccardo Faletti, Marco Gatti, Andrea Di Chio, Marco Fronda, Matteo Anselmino, Federico Ferraris, Fiorenzo Gaita, Paolo Fonio

**Affiliations:** 1 Radiology Unit, Department of Surgical Sciences, University of Turin, Via Genova 3, 10126 Turin, Italy; 2 Division of Cardiology, Department of Medical Sciences, “Città della Salute e della Scienza” Hospital, University of Turin, Turin, Italy

**Keywords:** Atrial fibrillation, Catheter ablation, Contrast media, Magnetic resonance imaging, Oesophagus

## Abstract

The purpose of this study was to compare *in vitro* pineapple juice and a solution of concentrated pineapple juice with a paramagnetic contrast agent in order to determine the feasibility of using the solution of concentrated pineapple juice *in vivo* for oesophagus visualisation at magnetic resonance angiography (MRA) before the radiofrequency catheter ablation procedure for atrial fibrillation. The pineapple juice was concentrated by a microwave heating evaporation process performed in a domestic microwave oven. Five grams of modified potato starch for every 40 mL of concentrated pineapple juice were added to the concentrated pineapple juice in order to thicken the solution. The solution resulted visually and quantitatively as hyperintense as the contrast agent *in vitro* (ratio = 1.02). *in vivo*, no technical difficulties were encountered during the MRA acquisition and a complete enhanced oesophagus was obtained in 37/38 patients (97.4%). The volumetric analysis and the three-dimensional reconstruction were feasible; the quality was rated as diagnostic in every patient. The intensified oesophagus was successfully merged into the electro-anatomical maps in all the patients. In summary, we demonstrated that this technique allows a feasible and safe oesophagus visualisation during MRA.

## Key points

- The imaging of the oesophagus may contribute to improving the safety of the radiofrequency catheter ablation procedure for atrial fibrillation.
- The concentrated pineapple juice resulted visually and quantitatively as hyperintense as the magnetic resonance contrast agent *in vitro*.
- The oesophagus was visualised and easily merged into the electro-anatomical maps in all the patients.
- A pre-procedural assessment based on the execution of a MRA with oral concentrated pineapple for visualisation of the oesophagus could represent the all-in-one pre-procedural imaging examination before radiofrequency catheter ablation for atrial fibrillation.

## Background

Radiofrequency catheter ablation (RFCA) of atrial fibrillation (AF) has become a common ablation procedure performed worldwide [1]. Asymptomatic ulceration or haemorrhagic thermal lesions of the oesophagus are seen in up to 15% of patients following AF ablation [2], this rarely leading to delayed fistula formation with the left atrium (LA). Although the reported worldwide incidence of atrial-oesophageal fistula as a complication of AF ablation is low (0.03–0.2%), it has a high mortality and morbidity rate [3].

Computed tomography angiography and magnetic resonance angiography (MRA) are the standard of care imaging techniques for LA and pulmonary vein visualisation [4–8] and the imaging of the oesophagus may contribute to improving the safety of procedures [4–7, 9–11].

Several studies considered different natural contrast agents to suppress or enhance the signal of the digestive tube [12–14]. Among them, there are two groups leading to either bright lumen (pineapple, blueberry juice) or dark lumen (tap water, orange juice) on T1-weighted images. Among the ones with a high signal on T1-weighted images, both Espinosa et al. [15] and Arthurs et al. [16] found pineapple to be the fruit that contains the highest concentration of Mn when compared with other fruits analysed *in vitro*. In addition, it is well known that during the concentrating process, the water is partially removed in the form of vapour from a boiling solution, while solid compositions such as vitamins, minerals and sugars do not change [17].

The purpose of this study was to evaluate the feasibility of the oesophagus visualisation with oral administration of a solution of concentrated pineapple juice during MRA.

## Methods

### *In vitro* study

#### Study design

**Fig. 1:**
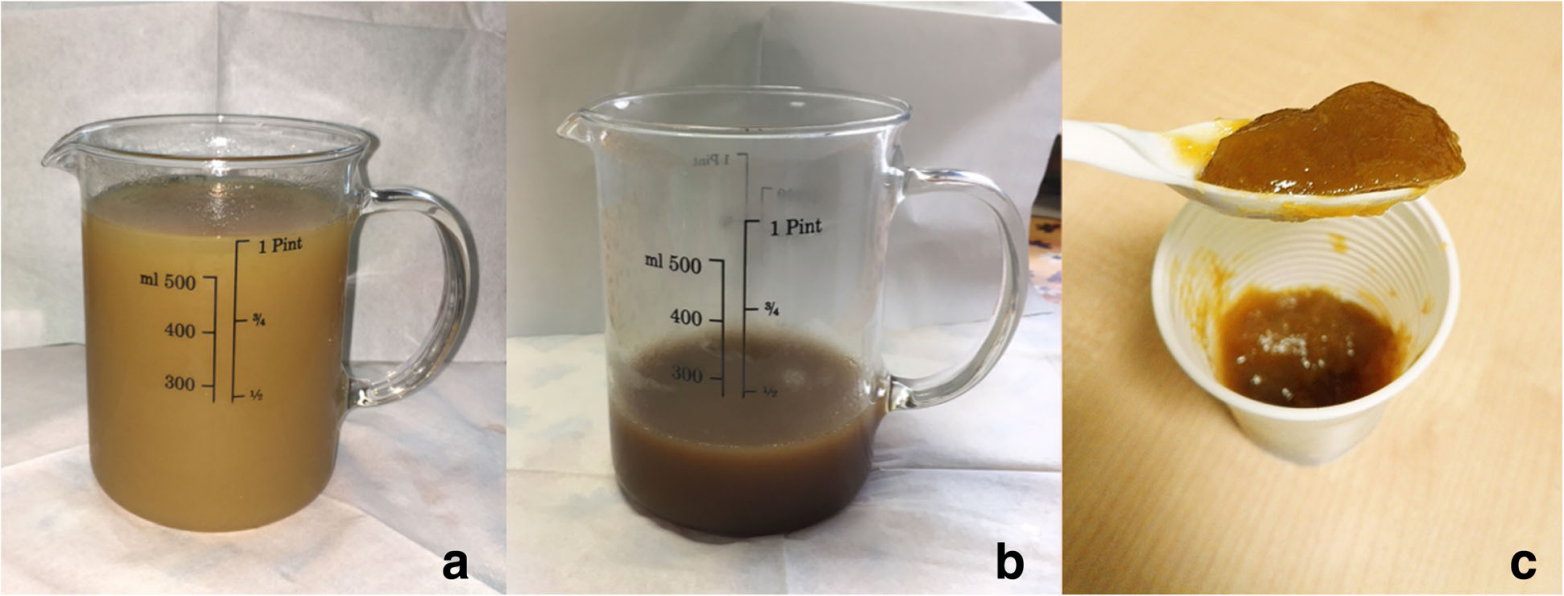
**a** Pineapple juice before the microwave heating evaporation process. **b** Concentrated pineapple juice after the microwave heating evaporation process. **c** The solution after the addition of modified potato starch

One pint (473.716 mL) of pineapple juice placed in a glass container was concentrated by a microwave heating evaporation process performed in a domestic microwave oven (MS11K3000AS, Samsung, Maetan-dong, Yeongtong District, Suwon, South Korea). The microwave’s power was set to 850 W for 25 min. Five grams of modified potato starch (Gel’M instantané ed. végétal, Nutrisens MEDICAL, Francheville, France) for every 40 mL of concentrated pineapple juice were added to the concentrated pineapple juice in order to thicken the solution (Fig. 1).

**Fig. 2:**
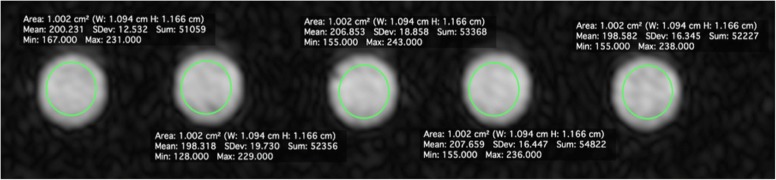
MRA axial images of the five 10-mL syringe filled concentrated pineapple juice plus modified potato starch

To evaluate the reproducibility of the microwave heating evaporation processes, we compared five 10-mL syringes filled with concentrated pineapple juice plus modified potato starch obtained from five consecutive and independent concentration process (Fig. 2).

**Fig. 3:**
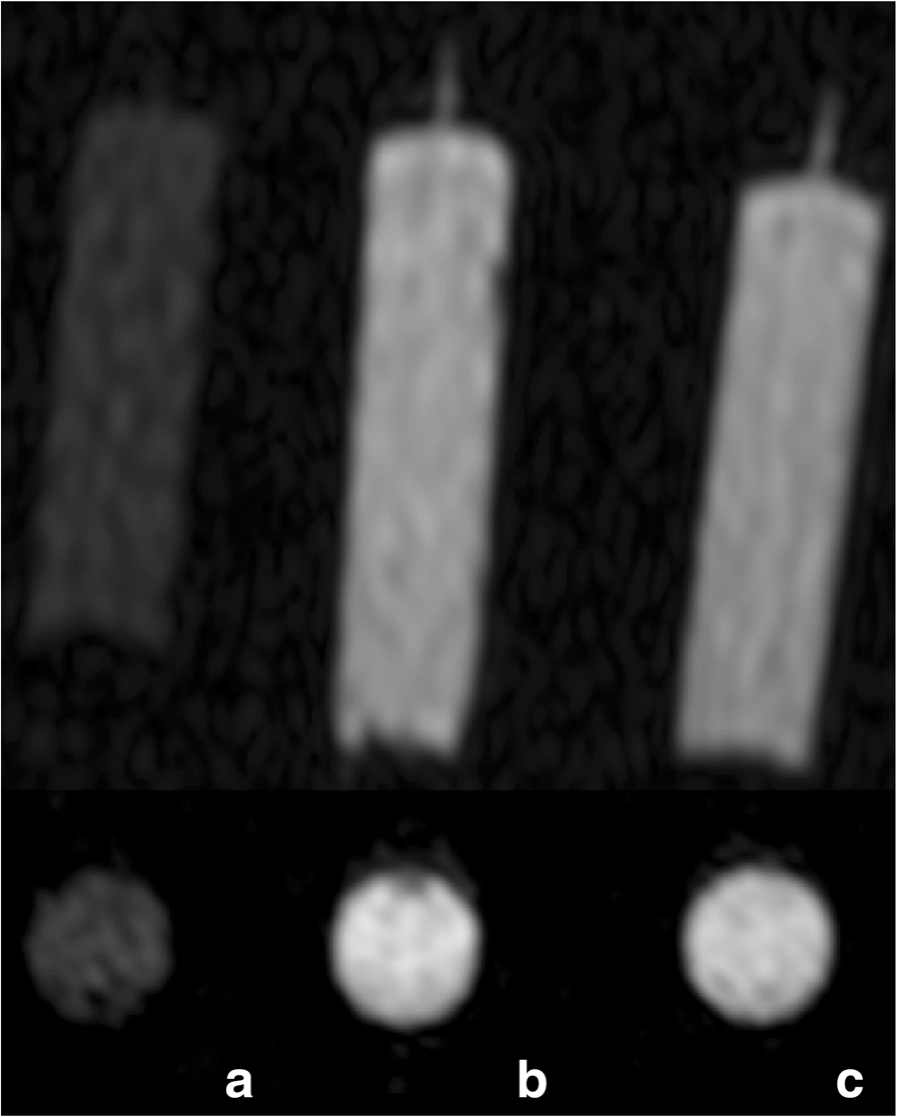
MRA axial and the corresponding coronal images of three 10-mL syringe filled with: (**a**) pineapple juice; (**b**) concentrated pineapple juice plus modified potato starch; and (**c**) saline diluted gadoteriol at a 3% concentration

To determine whether the solution so obtained retained the paramagnetic characteristics required by magnetic resonance imaging (MRI) contrast agent, we compared it to a contrast agent we routinely use for MRA (Fig. 3). We scanned three 10-mL syringes filled with: (1) pineapple juice; (2) concentrated pineapple juice plus modified potato starch; and (3) saline diluted gadoteridol (279.3 mg/mL, ProHance®, Bracco Altana Pharma, Constance, Germany) at a 3% concentration. This concentration was chosen based on the hypothesis that the contrast agent, after being injected at a flow rate of 2.5 mL/s, is homogeneously mixed with the blood pool on its arrival to the heart; therefore, for a mean cardiac output of 5 L/min (83.3 mL/s), its concentration is about 2.5/83.3 = 3%.

#### MRA protocol and image analysis

MRA was performed with a 1.5-T scanner (Achieva, version 2.6, Philips Medical Systems, Eindhoven, The Netherlands) using a 32-channel body phased-array coil. A three-dimensional (3D) spoiled gradient-echo sequence was acquired in the axial plane, with the following technical parameters: repetition time 3.3 ms; echo time 1.2 ms; flip angle 20°; right to left phase encoding; 512 × 512 matrix with an isotropic voxel of 1.5 mm^3^.

All MRA studies were analysed in consensus by two experienced observers with > 5 years of experience in cardiovascular MRI using OsiriX MD DICOM Viewer (Pixmeo Inc., Bernex, Switzerland).

To compare the intensity of the scanned syringe, a region of interest with a mean size of 1 cm^2^ was placed in the axial plane in the centre of each image (Figs. 2 and 3).

### *In vivo* study

#### Study design

The study was piloted in agreement with the 1964 Helsinki declaration and its later amendments and was approved by the ethics committee of our institution. Before MRA, all patients herein considered were informed about the possible use of their data for study purposes and gave their consent. Patient information was anonymised before the analysis.

Inclusion criteria were: symptomatic AF refractory to at least one anti-arrhythmic drug; age ≥ 18 years; and preserved left ventricular ejection fraction at echocardiography. Exclusion criteria were: active hyperthyroidism; impaired left ventricular function; pregnancy; previous oesophageal-gastric surgery; and contraindications to anticoagulation or MRA.

#### MRA protocol and image analysis

For the *in vivo* study, the same not electrocardiographically gated, free-breath sequence was performed after intravenous injection of 0.1 mmol/kg of gadoteridol at a rate of 2.5 mL/s, followed by a 20-mL saline bolus at the same rate. The mean sequence time was 25 s (range 19–32 s). Bolus tracking was used to start the sequence at the exact moment the contrast intensified during the venous phase of pulmonary circulation, to guarantee the maximum signal intensity in the pulmonary veins and into the LA. The oesophagus was intensified by administration of 40 mL of concentrate pineapple with 5 g of modified potato starch (Gel’M instantané ed. végétal, Nutrisens MEDICAL, Francheville, France), served with a disposable plastic spoon, while the patients were on the scanning table before the sequence acquisition.

After MRA, all patients were clinically monitored for 30 min. Any adverse effects or anomalies were registered after RFCA before discharge.

For the *in vivo* study, the feasibility of LA volume and left appendage volume calculation and the quality (classified as diagnostic or non-diagnostic) of 3D maximum intensity projection and 3D volume rendering reconstructions were evaluated to assess the spatial position of the oesophagus and identify appendage morphology and any anatomical variations of pulmonary veins.

## Results

### *In vitro* study

Among the five 10-mL syringes filled with concentrated pineapple juice plus modified potato starch, the ratio between the signal intensity measured in the syringe with the lowest signal intensity and the one with the highest was 198.138 / 207.659 = 0.95 (Fig. 2). The ratio between the signal intensity measured in the syringe filled with pineapple juice and the one with diluted gadoteriol was 59.508 / 198.361 = 0.30, while the ratio between the signal intensity of the syringe filled with pineapple juice with the addiction of modified potato starch and the one with diluted gadoteriol was 202.329 / 198.361 = 1.02 (Fig. 3).

### *In vivo* study

Forty patients (35 men, mean age 59 years) who met the above-mentioned criteria were enrolled in the study. In all patients, MRA was performed within 24 h before RFCA. In our series, we had to skip the administration of the oral solution in only two patients, due to known history of severe dysphagia and diabetes. No technical difficulties were encountered during all the MRA acquisitions.

We obtained a complete enhanced oesophagus in 37/38 patients (97.4%); in the remaining patient, the oesophagus was partially enhanced due to peristaltic waves.

**Fig. 4:**
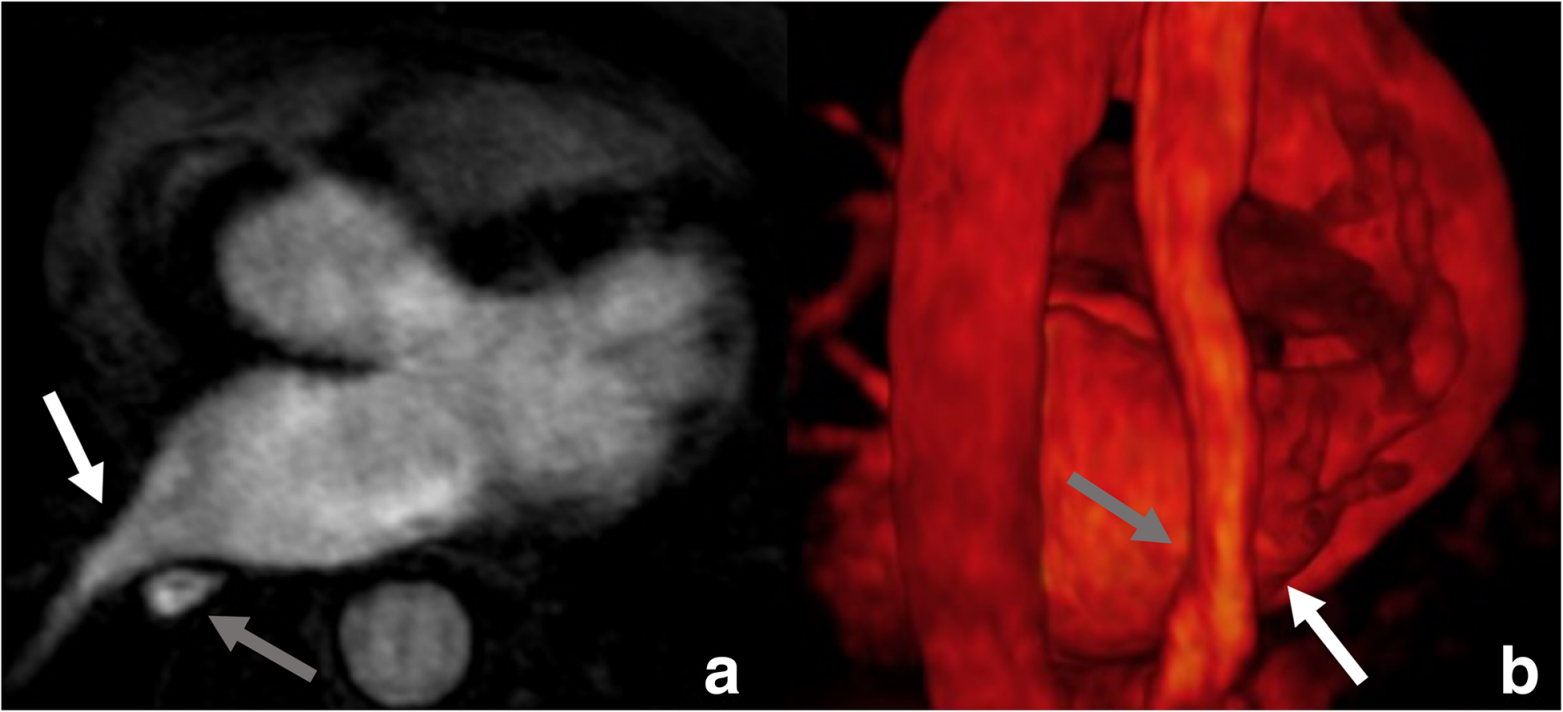
Axial images (**a**) with the corresponding 3D volume rendering (**b**) of the MRA. Note how the oesophagus (*grey arrow*) courses near the ostia of the right inferior pulmonary vein (*white arrow*)

The analysis of LA volume and left appendage volume was feasible in every patient. The quality of 3D maximum intensity projection and volume rendering reconstruction (Fig. 4), to assess the spatial position of the oesophagus and identify appendage morphology and any pulmonary veins anatomical variations, was rated as diagnostic and the oesophagus was visualised and merged into the electro-anatomical maps generated with Carto™ (Biosense Webster, Diamond Bar, CA, USA) in all patients. No immediate or late complication were developed in these patients. In particular, no clinical oesophageal symptoms were observed.

## Discussion

Our study demonstrated that the oral administration of a solution of concentrated pineapple juice for the oesophagus visualisation is a reliable and feasible technique without side effects.

Pineapple juice is widely used as an oral negative contrast agent in MRI cholangiopancreatography [18, 19], sometimes with the addition of gadopentetate dimeglumine [13, 20], due to its relatively poor Mn concentration. On the other side, it is used as an oral positive contrast agent, essentially in the real-time MRI of swallowing [21, 22].

The microwave heating evaporation processes herein presented allowed us to obtain, after a few attempts, an appropriate concentrate solution that was as hyperintense as a diluted contrast agent. This is fundamental for obtaining an optimal image quality *in vivo*, to allow image-reformatting approaches (e.g. 3D reconstruction with variable thickness viewing) and other post-processing methods, as well as the integration of the images into the electro-anatomical maps.

To the best of our knowledge, this is the first experience with the use of a ‘natural’ concentrated MRI contrast agent. Our group [7] first demonstrated the feasibility of the oesophagus visualisation with oral gadobenate dimeglumine during MRA. In that study, the oesophagus was enhanced using an oral gel solution of 0.7-mL gadobenate dimeglumine contrast agent mixed with 40 mg thickened water gel, which was swallowed by the patients on the scanning table, immediately before the MRA sequence acquisition. With our protocol, we now obtained the same results without the gadobenate dimeglumine.

In Europe, the use of macrocyclic agents (gadobutrol, gadoteric acid, and gadoteridol) can continue to be used in their current indications, but in the lowest doses that enhance images sufficiently and only when unenhanced body scans are not suitable [23]. Even if the orally administered Gd-based contrast agent was excreted in almost all the faeces and not absorbed [24], the use of concentrated pineapple juice seems to be a good way of reducing administered dose of contrast media.

Starek et al. [25] reported in their paper that failed oesophagus visualisation was most often related to delayed swallowing of the contrast agent or rapid passage through the oesophagus; therefore, as previously done by our group [7], we first had used thickened gel water, commonly used safely in patients suffering from a swallowing dysfunction and airway problems, and then we used modified potato starch to thicken the concentrated pineapple and with this method we prolonged the oesophageal transit time and allowed the patient to swallow it a few seconds before the administration of contrast agent.

Some studies showed a relatively stable position of the oesophagus in the long term [6, 26]. Conversely, other studies [27, 28] found a poor correlation between the pre-procedural view of the oesophagus and its actual position during the ablation procedure. Recently it has been proven that there are no significant movement of the oesophagus in the short term [25]. Based on this consideration, on the fact that CTA and MRA provide similar information before RFCA [29] and that the contrast-enhanced sequences seem to be highly sensitive and specific for diagnosing LA or LA appendage thrombus [30], a pre-procedural assessment based on an MRA with oral administration of concentrated pineapple for visualisation of the oesophagus a few hours before RFCA could represent the all-in-one pre-procedural imaging examination.

This study has some limitations. First, it is a single-centre study which covers a limited number of patients. Second, we did not directly compare this technique to the similar one based on the administration of a gel solution of gadobenate dimeglumine [7]. Third, we did not do a quantitative analysis *in vivo*; however, the fact that all the MRA were considered as diagnostic and we were able to merge the imaging into the electro-anatomical maps in all patients, makes us confident of the excellent quality of the *in vivo* images.

In conclusion, the pineapple juice after an appropriate concentration process and the addiction of modified potato starch is as hyperintense as the MRI diluted contrast media and allows a feasible and safe oesophagus visualisation during MRA.

## Abbreviations

3D: Three-dimensional
AF: Atrial fibrillation
LA: Left atrium
MRA: Magnetic resonance angiography
MRI: Magnetic resonance imaging
RFCA: Radiofrequency catheter ablation
